# Survival rate of teeth with periodontally hopeless prognosis after therapies with intentional replantation and perioprosthetic procedures ‐ a study of case series for 5–12 years

**DOI:** 10.1002/cre2.25

**Published:** 2016-03-23

**Authors:** Guey‐Lin Hou, Lein‐Tuan Hou, Arnold Weisgold

**Affiliations:** ^1^ Graduate Institute of Dental Science, Department of Periodontal Prosthesis, College of Oral Medicine Kaohsiung Medical University Kaohsiung Taiwan; ^2^ Graduate Institute of Dental Science, Department of Periodontics, School of Dentistry National Taiwan University Taipei Taiwan; ^3^ Graduate Institute of Periodontal Prosthetics, School of Dental Medicine University of Pennsylvania Philadelphia PA USA

**Keywords:** Ankylosis, ATP, bone fills, CSCTD, extensive bone loss, IR, periodontal prosthesis, PHP, root resorption, SOT

## Abstract

The purpose of the present study was to evaluate the longitudinal survival rate of the treatment of teeth affected with periodontally hopeless prognosis and secondary occlusal traumatism (SOT) using intentional replantation (IR) and periodontal prosthesis. We collected data from 17 individuals who received IR and participated in the study during 1995 to 2014. Of the 17 teeth affected by periodontally extreme conditions with deep angular bone defects, severe alveolar bone loss extending to or beyond the apex, and SOT, was recognized as having hopeless prognosis. Those teeth were treated sequentially using procedures that included basic periodontal therapy, therapeutic provisional prosthesis, IR, fixed dental prosthesis, crown and sleeve‐coping telescopic dentures (CSCTDs), or fixed prosthesis and CSCTD combined. Longitudinal assessments of clinical parameters and radiographic bone change before and after IR were evaluated. Clinical results showed that the overall cumulative survival rate of assayed teeth after IR therapy (5–12 years) was 88.2%. The mean (**±**SD) estimated radiographic alveolar bone loss was 12.7 ± 2.1 mm (88.5% ± 13.3%) of the root length, initially, and estimated radiographic alveolar bone gain was 4.0 ± 2.2 mm ultimately, in 17 replanted teeth with SOT. Only one tooth (5.9%) exhibited root resorption. Ankylosis was not observed during the study. Periapical radiographs demonstrated that satisfactory periodontal healing of lamina dura and bone fills occurred in all replanted teeth with SOT. Generally, tooth mobility and SOT were significantly improved after therapy. Most treated teeth functioned well and remained stable clinically throughout the periods of study. The present study documented a promising outcome for autogenous IR and periprosthetic therapy of 17 periodontally hopeless teeth for 5–12 years. The present study revealed good bone gain and elimination of SOT and prominent occlusal function. We concluded that the application of IR, minocycline‐HCL and periodontal prosthetic procedures later elevated the prognosis of these otherwise hopeless teeth with SOT, which are valuable options for retaining teeth with periodontally extreme situations.

## Introduction

Autogenous transplantation (ATP) has been previously recommended as a technique for use in various dental disciplines – such as prosthodontics (Tsukiboshi 2012), orthodontics (Andreasen et al. 1990; Tsukiboshi 1997, 2012), endodontics (Grossman 1966, 1980), and periodontics (Baer & Gamble, 1966). Tsukiboshi (2001) classified ATP into three groups: conventional transplantation, intraalveolar transplantation, and intentional replantation (IR). Baer and Gamble (1966) proposed ATP as a method for treating osseous defects in periodontosis with adequate attached periodontal ligament tissue. Grossman (1966, 1980) reported that IR could be regarded as a form of transplantation.

Intentional replantation is used as an alternative treatment option for hopeless teeth when conventional therapies have a high probability of difficulty or failure (Messkaub 1991). It should also be considered as a viable method particularly in efforts to preserve natural dentition. IR is mostly used for the treatment of lesions of endodontic origin, apicoectomy (Grossman 1966, 1980), surgical extrusion for deep decay (Tsukiboshi 1995, 1997), and cases of suspected root fractures (Tsukiboshi 2012, Grossman 1966, 1980).

Patients having severely advanced periodontitis (SAP) (bone loss ≥ 60%) and secondary occlusal traumatism (SOT) with a guarded prognosis in studies of periodontal and prosthetic therapies were found to be more prone to further alveolar bone loss (ABL) because of tooth hypermobility, pathological migration, and unpredictable outcomes of prosthetic abutments and eventually required extraction. Furthermore, even series of periodontal therapy including flap operation, guided tissue regeneration (GTR), GTR with bone grafting, application of enamel matrix derivative (EMD) (Tonetti et al. 2002), or their combined approaches still pose significant complications and discrepancies in success rates for cases of SAP with SOT.

There have been a few recent reports regarding successful treatment with IR, or IR and platelet‐rich plasma in cases with periodontally hopeless prognosis (Demiralp et al. 2003; Tözüm et al. 2006). Nevertheless, these studies were evaluated, only on the basis of limited case reports from short‐term and mid‐term periods. Recently, Baltacioglu et al. (2011) replanted 12 periodontally hopeless teeth using a combination of EMD and demineralized freeze‐dried bone allografts (DFDBA) in treating extensive ABL, vertical bone defects, and severe ABL extending to the apexes. They concluded that IR combined with a local mediator and bone graft could be a successful procedure for saving teeth while monitoring with clinical and radiographic evaluations for 12 months after therapy.

Exciting clinical results in regenerative periodontal therapy in hopeless teeth, presenting extensive bone loss at or beyond the root apex, have been recently reported (Cortellini et al. 2011). In their study, regeneration therapy led to retention of 92% of the teeth scheduled for extraction and improved their final prognosis and comfortable functioning. However, only 5 years of observation was reported. Further clinical follow‐up data are necessary to monitor the long‐term stability of bone fill and periodontal parameters.

Little longitudinal documentations related to combination of IR/crown and sleeve‐coping telescopic denture (CSCTD) treatment of periodontally hopeless teeth in cases with SAP and SOT has been reported (Hou et al. 1997, 1999). The purposes of this study were to explore and longitudinally evaluate the outcome of a treatment using a periodontal prosthetic after replantation of teeth under extreme periodontal conditions.

## Materials and Methods

### Collection of teeth

Seventeen teeth from 17 Taiwanese subjects (ranging 35–65 years in age), were diagnosed as having SAP with SOT and extensive ABL between 1995 and 2014. Clinical and radiographic examinations revealed gingival inflammation accompanied with pus discharge at some tooth sites, extensive ABL, grades II to III tooth mobility, and SOT on affected teeth usually. These teeth required extraction for periodontal treatment, and implant therapy was suggested by local dentists, but refused by patient.

All patients were in good general health with no contraindications for receiving dental treatment. Radiographic assays, for ABL and alveolar bone gain of replanted teeth, were carried out using projected radiographic image analysis (Lin et al. 1996). Lamina dura, widening of periodontal ligament space, and the degree of angular bony defects were evaluated at baseline, 3 months, 6 months, and throughout the individual periods of study from 1995 to 2014. The remaining number of teeth, gender, age, amount of ABL and ABF, treatment and follow‐up periods, and survival rates are listed in Table 1.

**Table 1:** Demographic data at baseline and survival rate with periodontally hopeless prognosis after intentional replantation therapy.

Cases	Tooth Location	Age	Baseline				Final			Tooth	Tooth
			RABL (%)	RABL (mm)	RBH (mm)	RRL (mm)	RBH (mm)	RRL (mm)	RBG (mm)	F‐U (ms)	S/L
Case 1	#13	53	100	16.0	<0*	14.8	7.9	12.3	7.9	80	S
Case 2	#11	65	92	15.7	1.3	17.0	7.0	9.7	5.7	93	S
Case 3	#14	41	97	14.1	0.5	14.6	3.3	10.0	2.8	112	S
Case 4	#45	43	100	15.3	0	15.3	2.7	8.0	2.7	105	S
Case 5	#34	42	88	11.4	1.6	13.0	4.0	10.0	2.4	99	S
Case 6	#22	43	100	14.0	0	14.0	3.0	9.3	3.0	98	S
Case 7	#22	42	83	14.0	2.8	16.8	3.8	9.0	1.0	144	L
Case 8	#21	55	80	11.4	2.9	14.3	6.7	9.0	3.8	84	L
Case 9	#21	35	100	12.3	0	12.3	2.0	13.0	2.0	120	S
Case 10	#24	44	100	11.3	<0*	11.0	5.3	10.0	5.3	70	S
Case 11	#11	56	85	13.4	2.3	15.7	8.0	13.3	5.7	66	S
Case 12	#11	42	60	9.0	6.0	15.0	8.7	12.0	2.7	64	S
Case 13	#36	51	100	12.0	0	12.0	6.7	9.0	6.7	63	S
Case 14	#35	53	70	10.0	4.3	14.3	6.0	8.0	1.7	74	S
Case 15	#12	53	83	13.3	2.7	16.0	4.7	12.7	2.0	61	S
Case 16	#25	51	100	13.3	0	13.3	4.0	7.0	4.0	70	S
Case 17	#25	51	66	9.7	5.0	14.7	13.3	13.3	8.3	60	S
Total	17	17	17	17	17	17	17	17	17	17	15S/17
Mean (SD)		48.2 (7.5)	88.5 (13.3)	12.7 (2.1)	1.7 (2.0)	14.4 (1.6)	5.7 (2.8)	10.3 (2.0)	4.0 (2.2)	86.1 (24.1)	
Median		51	92	13.3	1.3	14.6	5.3	10	3.0	80	
Survival rate											88.2%

Data obtained from 1995 to 2014 with a follow‐up period of 60–144 months.

< 0*, radiographic bone loss beyond root apex; RABL (mm), radiographic alveolar bone loss; RABL(%), RABL (mm)/RRL (mm) × 100%; RBH (mm), radiographic bone height; RRL (mm), radiographic root length; F‐U, follow‐up (months, ms); S/L (%), replant survival (n)/replant loss × 100%; BRG (mm), radiographic bone gain; RBG, final RBH‐baseline RBH; SD, standard deviation.

**Figure 1:**
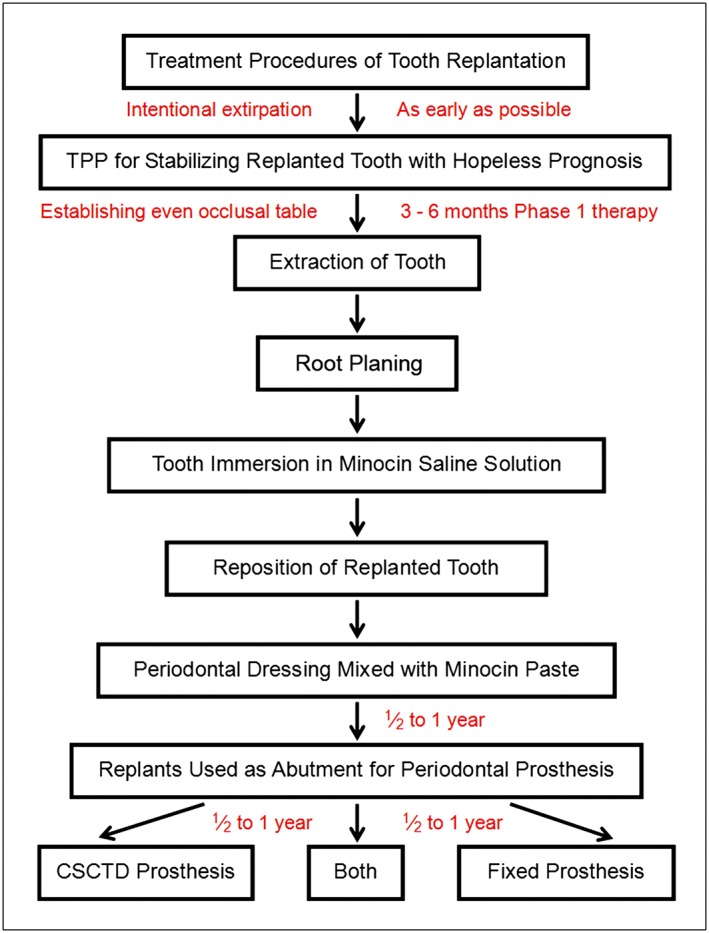
Flow chart of replantation procedures. Therapeutic provisional prosthesis (TPP); crown and sleeve‐coping telescopic denture (CSCTD).

Patients were regularly treated with a series of phase I periodontal therapies including oral hygiene instruction, scaling/root planing, and pocket irrigation with 0.1% chlorhexidine gluconate (Scodyl‐F, cGMP; Washington Pharmaceutical Co., Taiwan) solution after informed consent was given. Intentional pulp therapy, IR, periodontal prosthetic procedures, and establishment of the vertical dimension and immobilization of replanted teeth (splinted therapeutic provisional prosthesis, TPP) were performed sequentially. The flow chart in Figure 1 illustrates IR and other therapeutic procedures.

Clinical and radiographic evaluations after therapies, including SOT, root resorption, tooth mobility, changes of bone defects, incidence of ankyloses, and types of prosthetic design, were recorded every 3–6 months until the end of the study (Table 2).

**Table 2:** Clinical parameters including SOT, root resorption, mobility, ankylosis, and types of prosthesis design in teeth affected with periodontally hopeless prognosis before and after intentional replantation.

Cases No.	Tooth location	SOT		Root	Mobility (grade)		Ankylosis	Prosthesis design		
		Baseline	After	Resorption	Baseline	After		FP	CSCTD	Both
Case 1	#13	Yes	Imp	N	III	I	N		Y	
Case 2	#11	Yes	Imp	N	III	I	N	Y		
Case 3	#14	Yes	Imp	N	III	I	N	Y		
Case 4	#45	Yes	Imp	N	III	II	N	Y		
Case 5	#34	Yes	Imp	N	III	II	N			Y
Case 6	#22	Yes	Imp	N	III	II	N			Y
Case 7	#22	Yes	Imp	Y	III	II	N	Y		
Case 8	#21	Yes	Imp	N	III	II	N			Y
Case 9	#21	Yes	Imp	N	III	II	N	Y		
Case 10	#24	Yes	Imp	N	III	II	N		Y	
Case 11	#11	Yes	Imp	N	III	I	N			Y
Case 12	#11	Yes	Imp	N	II	I	N	Y		
Case 13	#36	Yes	Imp	N	II	I	N	Y		
Case 14	#35	Yes	Imp	N	III	I	N			Y
Case 15	#12	Yes	Imp	N	III	II	N	Y		
Case 16	#25	Yes	Imp	N	III	II	N	Y		
Case 17*	#25	N	N	N	II	I	N	Y		
Total	17	16/17	16/16	1/17	15‐III/2‐II	9‐II/8‐I	0/17	10/17	2/17	5/17
%		94	100	5.9	100	100	100	59	11	30

SOT, secondary occlusal trauma; Y, yes; Imp, improved; N, no; FP, fixed prosthesis; CSCTD, crown and sleeve‐coping telescopic denture; Both, FP and CSCTD; Case 17*, tooth #25 with symptoms of periapical lesion and sinus tract, but no SOT during permanent periodontal prosthesis.

### Results

Seventeen replanted teeth (13 maxillary and four mandibular) were included in the present study. Nine maxillary replanted teeth were located in the anterior region, while the other seven were premolars on both jaws. One molar with a mesial root resection was on the lower jaw (case 13). The mean periods of observation were 86.1 ± 24.1 months (range, 60–144 months; median, 80 months) after IR therapy. The mean age of the patients was 48.2 ± 7.5 years (range, 35–65 years; median, 51 years) at baseline (Table 1).

**Figure 2:**
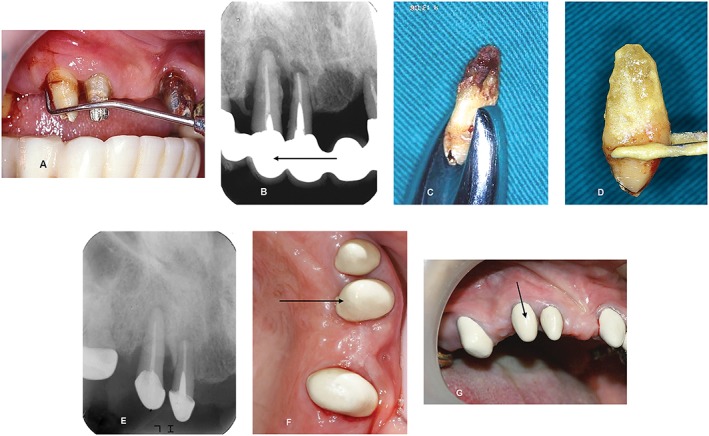
(A) Clinical probing depth >10 mm was found at the distopalatal area of tooth #13. (B) Complete bone loss was extended to the root apex of teeth #13 and #12. (2007) (C) Heavy calculus deposits were noted surrounding the apical third after extraction of tooth #13. (D) Immersion of tooth #13 in minocycline‐HCL paste for 5 min after thorough scaling and root planing. (E) Periapical radiograph illustrated a large amount of bone regeneration around the replanted tooth #13 in 2012. (F) Clinical picture in 2014 indicated an improvement of periodontal tissue. (G) Clinical picture in 2014 showed a stable and healthy periodontal tissue around the replanted tooth #13 for 80 months after complete reconstruction of inner crown to serve as one of the abutments for crown and sleeve‐coping telescopic denture.

The mean radiographic ABL (% and mm) before IR was 88.5% ± 13.3% (range, 60–100%; median, 92%) and 12.7 ± 2.1 mm, respectively, at baseline. Radiographs taken at short‐term, mid‐term, and long‐term follow‐ups were recorded after IR. Table 1 also showed that 4.0 ± 2.2 mm alveolar bone gains (median, 3.0 mm) were observed at the end of follow‐up periods for a mean of 86.1 ± 24.1 months (5–12 years) and a median of 80 months. Representative clinical and radiographic pictures in case 1 showed stable and healthy periodontal tissue around replanted tooth #13 for 80 months after complete reconstruction of the inner crown to serve as one of the abutments for CSCTD restoration (Fig. 2F, G). In addition, 7.9 mm of bone gain was achieved 80 months following IR even through there was an almost complete bone loss initially (Fig. 2B and Table 1).

The mean survival rate of the 17 replanted teeth after experimental therapies was 88.2% (15/17) during 5‐ to 12‐year follow‐up periods (from 1995 to 2014) (Table 1). Two replanted teeth were lost at 144 and 84 months (cases 7 and 8), respectively. One of the 17 replanted teeth (#22, case 7) was extracted after 12 years of functioning because of root resorption, an incidence of 5.9% (1/17 ratio) (Table 2). Tooth #21 (case 8) was lost because of periodontal complications with diabetes mellitus and poor hygiene. Ankylosis was not found in any cases radiographically during the entire follow‐up periods.

The types of periodontal prosthesis used following successful IR therapy (3–6 months postoperatively) included fixed prosthesis (FP) (10 cases, 59%), CSCTD (two cases, 11%), and combinations (five cases, 30%) (Table 2). Before final restoration, TPPs were routinely used in the present study to stabilize the teeth, for at least for 6–12 months for the management of hypermobile replanted teeth with SOT because of them having less periodontal bony support, and plus a healing period after the IR procedure.

## Case Reports

### Case 1

A 53‐year‐old woman (2007) presented at our office seeking treatment for tooth #13 with primary complaints of recurrent gingival swelling, bleeding with pus discharge, poor esthetics, and difficulty in chewing because of teeth hypermobility. She had visited many local dentists for treating her periodontal problems and been told that all mobile teeth should be extracted, and dental implant therapy was suggested. However, she rejected those treatment plans and showed strong motivation to keep the teeth (Fig. 2).

Oral examination showed that moderate to severe gingival recession and swelling were generally found on the remaining teeth. Initial probing of tooth #13 revealed deep pockets (7, 8, and 9 mm) buccally as well as (8, 8, and >10 mm) palatally, with gingival recession of 2 mm at the mesiopalatal site (Fig. 2A). Assessments of periodontal parameters of the remaining teeth included gingival index (GI) (Löe and Silness 1963), plaque index (PlI) (Silness and Löe 1964), mobility test (Miller et al. 1957), probing pocket depth (PPD), and clinical attachment level (CAL), being recorded at baseline and every 6 months until the end of the study.

Periapical radiographs disclosed that a severe ABL with deep angular bony defects and SOT were noted around teeth #13 (ABL > 100%) and #12. Complete bone loss extending over the root apex of teeth #12 and #13 was also found (Fig. 2B). Mobility of grades II and III was noted in most of the remaining maxillary teeth after removal of ill‐fitted prosthesis on teeth #12, #13, #17, #21, and #22. A diagnosis of generalized chronic periodontitis with SOT was established. Treatment plans including basic periodontal therapies, fixed TPP, IR and later periprosthetic procedures (CSCTD, FP, or both) were proposed. The unpredictability of the prognosis and probable survival rate of the experimental therapeutic approach was explained to the patient.

The patient was subjected to a thorough plaque control program following routine subgingival scaling and root planing. Chlorhexidine gluconate (Scodyl‐F mouth fresh solution [0.1%]; cGMP Washington Pharmaceutical Co., Taiwan) was used for pocket irrigation at teeth #12 and #13 following the discussed procedures earlier. Subsequent recalls, for monitoring and reinforcement of oral hygiene, were established every 2 weeks for 6 months. Regular scaling was then conducted during later follow‐ups.

Therapeutic provisional prosthesis procedures for full‐mouth reconstruction were made to establish vertical dimensions and resolve SOT. The extraction of tooth #13 was performed atraumatically after preparing the replantation site under local anesthesia. Extracted tooth #13 showed that heavy calculus deposits surrounded the middle half and extended to the root apex (Fig. 2C). Thorough extraoral scaling and root planing were carried out. The prepared root surface was immersed in a paste mixture of minocycline‐HCL capsule (powder, 100mg; China Chemical and Pharmaceutical Co., Taiwan) and normal saline for 5 min (Fig. 2D). The minocycline‐HCL paste was freshly prepared for each tooth before replanting (modified procedure, Demiralp et al. 2003). Root‐conditioned tooth #13 was then replaced into its original socket and immobilized by cementation of prefabricated TPPs and Coe‐pack dressing.

Subsequent visits for postoperative care occurred every 6–8 weeks until the end of study. Clinical assessments, in the first 18 months, on tooth #13 after IR revealed remarkable improvement in periodontal parameters GI, PlI, CAL, PPD, and mobility (data not shown). Probing depths of replanted tooth #13 were reduced to normal ranges, except for a depth of 5 mm found at mesio‐palatal aspect. A specially designed permanent CSCTD (Hou et al. 1999) was constructed six months later. The clinical mobility of tooth #13 was remarkably improved from grade III at baseline to only slight mobility (<grade I) 5 years later.

Radiographs in 2012 illustrated remarkable bone fill around tooth #13 (Fig. 2E), compared with baseline (Fig. 2B). Clinical photographs of tooth #13 in 2014 (Fig. 2F, G) also exhibited stable and healthy periodontal tissue following applications of TPPs, IR, and CSCTD for 80 months.

### Case 2

**Figure 3:**
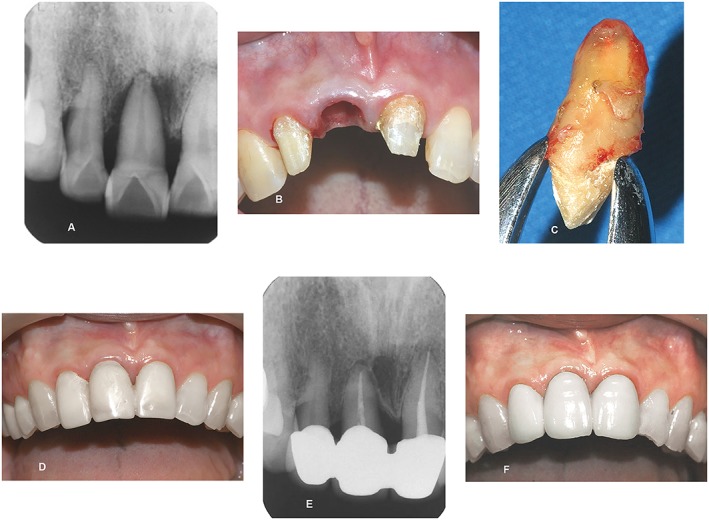
(A) Periapical radiograph illustrated a severe angular bony defect around tooth #11 (92%) periodontal bone loss. (B) Tooth #11 was extracted atraumatically. (C) Clean preparation of root surface on tooth #11 before its replantation into extracted socket. (D) Splinting therapeutic provisional prosthesis of teeth #12, #11, and #21 to cement the tooth back to its original sites. (E) Radiograph indicating the reappearance of the lamina dura, good bone fill, and periodontal healing in 2014. (F) Clinical picture in 2014 revealed healthy periodontal tissue at replanted tooth #11 and neighbor fixed prosthesis after functioning properly for 7 years and 9 months.

A 65‐year‐old woman was referred to Hou's clinic with chief complaints being recurrent gingival swelling, pus discharge, tooth sensitivity and mobility, and incapability of chewing with tooth #11. These symptoms had been observed off and on, since she was 60 years old. She visited a local dentist and was diagnosed as having localized, advanced periodontitis with SOT and extensive ABL around tooth #11. Extraction of tooth #11 and implant therapy was strongly suggested by local clinicians, but she refused and was referred to our office for further treatment (Fig. 3).

Periapical radiographs illustrated a generalized, severe angular bone loss at the anterior maxillary region. Angular defects around tooth #11 extended almost to the apical area with 92% bone loss at the mesio‐palatal site and PPD >10 mm (Table 1 and Fig. 3A). Grade III tooth mobility was detected on tooth #11. A diagnosis of localized, advanced periodontitis with SOT was made after clinical and radiographic examinations.

A conservative treatment plan of TPPs, IR, and permanent FP was prescribed and explained in detail to the patient. Periodontal parameters GI, PlI, CALs, and PPDs were measured (data not shown), a mobility test was recorded at baseline and every 6 months, and the ABL score was determined radiographically every 1–2 years, until the end of the study.

Basic periodontal therapies were performed similar to case 1. Pocket irrigation (0.1% chlorhexidine gluconate) was instituted during phase I periodontal therapy. Supportive periodontal therapy was established every 4–6 weeks for the initial 6 months. Splinting TPPs on teeth #12, #11, and #21 were constructed to immobilize tooth #11 suffering grade III mobility and SOT. The clinical response following phase I periodontal therapy was satisfactory except at the mesio‐palatal aspect of tooth #11, where a probing depth of 6 mm still existed.

Removal and sterilization of TPPs by dipping in the chlorhexidine gluconate solution (0.1%) were carried out. Tooth #11 was extracted atraumatically under local anesthesia (Fig. 3B). Meticulous scaling/root planing and removal of necrotic cementum using a high‐speed bur were then performed on extracted tooth #11 (Fig. 3C). The prepared root surface was conditioned by using fresh minocycline‐HCL (100 mg/mL) paste for 5 min. The extracted tooth was placed into its original socket and cemented to the splinted TPPs of teeth #12, #11, and #21 (Fig. 3D).

Finally, Coe‐pack dressing with minocycline‐HCL paste was applied. Minocycline‐HCL (100 mg) was applied twice daily for 5 days. Subsequent visits for monitoring wound healing were performed weekly around replanted tooth #11. The patient reported that she could chew normally one week later. Reinforcement of oral hygiene maintenance was established as before. Clinical and radiographic examinations of replanted tooth #11 revealed a healthy periodontal tissue, reappearance of lamina dura, and good bones fill (Fig. 3E) around the IR tooth at 7 years and 9 months (Fig. 3F).

## Discussion

### Clinical findings after intentional replantation and restorative procedures

Symptoms and signs that were exhibited before IR therapy disappeared almost completely within 1–2 weeks afterward. These results might reflect a benefit in that IR simultaneously offers complete accessibility of the root for planing purpose. Effective debridement was carried out at the apical root portion via an IR procedure, as opposed to a flap operation in which the root surface of a deep angular bone cannot be accessed easily.

### Regenerative technique

Recently, Baltacioglu et al. (2011) replanted 12 periodontally hopeless teeth in 11 patients using a combination of EMD and demineralized freeze‐dried bone allografts for treating extensive ABL, vertical defects, and periapical pathoses. They concluded that IR combined with regenerative procedures could be a successful strategy modality versus tooth extraction after a 12‐month follow‐up. The clinical application of IR without GTR on these periodontally hopeless teeth is not discussed in the literature.

The cumulative survival rate of replanted teeth was 88.2% (15/17) with a mean observation time of 86.1 ± 24.1 months (median 80 months). Our observations suggest an alternative option for teeth suffering from SAP and SOT, which are usually recognized as periodontally hopeless. Recently, excellent results from regenerative therapy for hopeless teeth, presenting with extensive bone loss at or beyond the root apex, achieved a 92% retention of treated teeth, improved final prognosis, and comfortable functioning in a 5‐year randomized clinical trial (Cortellini et al. 2011). Therefore, these treatment approaches provide a favorable environment for the early periodontal healing of soft and hard tissues and might contribute to the clinical success and long‐term survival of replanted teeth and favor the usage of the periodontal prosthesis.

### Survival rate

The majority of reports indicate that tooth replantation in humans is appropriate for cases of broken instruments, canal perforation or obturation, large periapical lesions, and tooth avulsion. In most situations, root resection is preferred to IR in molars (Grossman 1966).

In regard to the survival rate of IR, most published data evaluated observation periods and defined success criteria. Most of the older reports that discuss the survival rate of IR focused mainly on the treatment options of nonperiodontally involved hopeless teeth and various dental disciplines, such as prosthodontics, orthodontics, and endodontics. In contrast, few or no reports discuss the survival rate of IR in teeth affected with advanced bone loss and SOT under conditions of periodontally hopeless prognosis and the acceptance of prosthetic therapy in recent literature. The present 17‐case series mean accumulated survival rate was 88.2% (15/17) based on observations over 5–12 years (Table 1).

### Bone gain of extreme angular bone defects

Why do bone fills happen on periodontally hopeless teeth exhibiting SOT, and extensive ABL up to the root apex as in the present study? Andreasen (1981) suggested that even the loss of a periodontal ligament, up to 2 mm in width around the root surface, could be repaired by a new attachment. Similarly, remarkable healing of periodontal bone occurred after the IR procedure and up until the end of the study. Andreasen et al. (1990) further reported that successful periodontal healing occurred as evidenced by the reappearance of lamina dura and periodontal ligament space. In addition, these phenomena were completed within 2 months in most cases, although some cases having wider angular bone defects needed more time for healing. Optimal bone regeneration was also seen in a recent report of IR and GTR procedures using combinations of various graft materials and barrier membranes (Cortellini et al. 2011).

In fact, we found that the greater the distance between bone and root surface, the more time (cases 1, 2, and 11) was necessary for bone tissue healing to reach the root surface. Thus, a wide range of reparation of PDL and bone fill was needed in our study, even up to 5 years or more. Radiographically, the majority of replanted teeth in our study originally showed a remarkable loss of lamina dura, widening of PDL, and angular bony defects extending to the root apex; however, this still permitted prominent bone gain without bone grafting (e.g., cases 1 and 2). These findings are inconsistent with those of Andreasen and Kristerson (1981).

Histological studies on periodontal healing in animals (Tsukiboshi 2012; Andreasen 1981) indicate that periodontal repair associated concomitantly with root resorption was dictated by the presence of the cemented portion of the PDL and not the alveolar portion. Similar conclusions were found to be in agreement with Inoue et al. (1988, 1989) and Waikakul et al. (2011). They indicated that osteogenic activity within the periodontal ligament might regenerate bone around a donor tooth that has been replanted without enough supporting bone.

### Root resorption

An early report (Andreasen and Hjorting‐Hansen 1966) documented that 90% of teeth replanted within 30 min had no root resorption. Grossman (1966) also reported that 17.8% (8/45) of root resorption occurred 5.6 years after IR procedure. Only one (case 7) of the 17 replanted teeth having severe infrabony defects (bone loss 83%) in our study was extracted because of root resorption (1/17; 5.9%) after 12 years of observation.

### Root conditioning (antibiotics) and ankylosis

Recent reports have proposed that tetracycline‐HCL may play a role in the reduction of osteoclastic bone resorption and inhibition of collagenase activity during an early phase of healing (Keller & Carano 1995; Christersson et al. 1993; Demiralp et al. 2003). Additionally, conditioning of the root surface using minocycline‐HCL in the present study may have provided an antimicrobial effect, thus reducing the deleterious effects of an inflammatory response. This notion is supported by the fact that tetracycline is adsorbed and actively released from root dentin but still maintains bacteriostatic levels against most known periodontopathogens (Baker et al. 1983; Wikesjo et al. 1986). The elimination of microorganisms from root surfaces might decrease the frequency of ankylosis in replanted teeth (Cvek et al. 1990).

Interestingly, no ankylosis was found after IR therapy and throughout the follow‐up period of 5–12 years in this study. Tetracycline‐HCL‐treated root surfaces may improve substrates for connective tissues on the socket wall that are vital to healing at the interface between soft and hard tissues (Wikesjo et al. 1986; Christersson et al. 1993). The roles of an antibiotic application of minocycline‐HCL on the prevention of ankylosis and enhancement of periodontal healing during IR need to be further investigated.

### Immobilization and periodontal prosthesis associated with secondary occlusal trauma

Little information exists in the literature regarding the relationship between teeth with periodontally hopeless prognosis plus SOT after the application of IR and subsequent special periodontal prosthetic designs. The present study appears to be the first report to longitudinally evaluate the outcome of IR therapy, in which teeth affected with periodontally hopeless prognosis plus SOT received conservative reconstruction via periodontal prosthesis.

Mobility may affect pocket formation and apical migration of the epithelial attachment in the presence of inflammation and inhibit periodontal repair and bone regeneration during and after therapy (Demiralp et al. 2003). Stabilizing teeth with TPP, as an immobilization aid for hypermobile teeth, was part of the IR technique used with minocycline‐HCL application in the present study and found to be an effective therapy for periodontally hopeless prognosis teeth with SOT. Thus, splinting may give replanted teeth a chance to heal and reattach to soft tissue and bone in order to survive and favor subsequent tissue maturation and stability during follow‐up periods.

Previous studies (Yalisove 1966; Yalisove and Dietz 1977; Silverman 1986; Hou et al. 1997, 1999) also concluded that the use of FP, CSCTD, or combinations of both strategies provided a beneficial force distribution in cases with periodontally hopeless prognosis and SOT, especially in the shift of loading force to the long axis of the tooth and a minimization of horizontal forces. Even in cases when the abutments of CSCTD were lost, the edentulous area was easily restored prosthetically. Therefore, the present treatment using IR and a special prosthesis not only achieved a significant improvement in periodontally hopeless teeth with SOT but also provided an effective nonsurgical approach in these compromised situations.

### Tooth preservation or implant therapy

Over the past 30 years, implant therapy has been recognized as a safe and reliable method of treating patients with missing teeth. Owing to the general popularity and high survival rates of dental implants, dental practitioners usually believe that they are as good as natural teeth. This often resulted in the extraction of teeth that may be salvageable. Faced with the option of retaining a compromised tooth or placing a dental implant, the clinician should make an evidence‐based decision, rather than based on treatment convenience (Levin and Halperin‐Sternfeld 2013). Because an implant can serve as a replacement for an extracted tooth at any point, regardless of the length of time, the tooth had been maintained. However, tooth extraction is an irreversible treatment. The lack of solid information in the literature had been overtly addressed regarding the benefit and success of implants versus treatment outcomes of periodontally compromised teeth.

A recent critical review provides a valuable insight in this issue (Levin and Halperin‐Sternfeld 2013). This report assessed the long‐term survival rates and treatment outcomes for retained compromised teeth in comparison with the long‐term survival rates for dental implants. They found that the rate of tooth loss after periodontal treatment and during the maintenance phase was found to vary between 3.6% and 13.4% over the follow‐up periods ranging from 16 to 30 years. Overall, between 1.4 and 3.6 teeth per patient were lost. A similar incidence of tooth loss was noted in patients during supportive periodontal therapy, ranging from 3.8% to 11.3% after 16 to 22 years of follow‐up.

Their analysis also disclosed that even the tooth loss rates were low in those included studies (Levin and Halperin‐Sternfeld 2013); tooth extraction was often caused by a clinician's subjective opinion, which was not always indicative of the tooth's inability to survive in the long term. Interestingly, most studies included in this investigation, which assessed tooth survival, were conducted in periodontally compromised patients. Even this relatively low rate of tooth loss might still be an overestimation of the periodontitis‐related tooth loss rate, as reported by Hirschfeld and Wasserman (1978) and McFall (1982) and thus indeed an underestimation of the actual tooth survival.

Regarding the long‐term implant survival, the percentage of implant losses, reported in this review (Levin and Halperin‐Sternfeld 2013), varied between 0% and 33.6% during the follow‐up period of 15 to 20 years. The cumulative survival rate ranged between 69.6% and 100%, and the amount of bone loss varied between 0.05 and 2.1 mm. Studies with longer follow‐up periods (up to 23 years) reported a further bone loss that was twice as large as that reported in the study with a shorter period. These findings might contribute to a greater implant loss rate over longer follow‐up periods. The effectiveness of periodontal therapy and long‐term maintenance (supportive periodontal therapy) in preventing tooth loss in patients with severe periodontal disease has been unequivocally addressed in many studies (Hirschfeld and Wasserman 1978; McFall 1982; McGuire and Nunn 1996; Chambrone et al. 2010; Lindhe and Pacey 2014). In addition, one should keep in mind that the survival of an implant does not necessarily mean the treatment has been successful. Dental implants are not the reference standard for replacing compromised teeth because they will not survive forever (National Institute of Health Consensus Development Conference Summary 1978).

The belief of implants yielding a better long‐term prognosis has also clearly been questioned in the latest literature (Giannobile and Lang 2016). Teeth even compromised because of several periodontitis or endodontic problems may still have a longevity that surpasses that of the average implant (Lang and Zitzmann 2012; Salvi et al. 2014; Klinge et al. 2015). These data might suggest a generally higher rate of implant loss than of tooth loss, which supports the conclusion of Holm‐Pedersen and colleagues (2008) that implant survival will not surpass tooth survival over the long term. In light of the aforementioned evidence, the decision to preserve properly treated and maintained teeth for as long as possible seems to provide an overall solution that can reduce the treatment risks over the long term.

## Conclusions

The present study demonstrated promising outcomes (tooth survival rate of 88.2%) using autogenous IR therapy and perioprosthetic reconstruction of 17 teeth with periodontally hopeless prognosis with SOT during 5–12 years of observations. The present study demonstrated significant (or remarkable) periodontal bone gain, effective reduction of SOT, and maintenance of healthy periodontal tissues and functions in treated teeth throughout the study. The observations of our study and the latest message in literature may urge an action to revisit the long history of success of tooth maintenance to retain the natural dentition without the rush to extract teeth and replace with implants. We, thus, conclude that the combined use of IR and periodontal prosthetics could be an effective, valuable strategy for preserving teeth affected with periodontally hopeless or compromised situations.

## Conflict of Interest

None declared.

## Funding Information

This study has been self‐funded by the authors and their institutions.
